# MDM2 prevents spontaneous tubular epithelial cell death and acute kidney injury

**DOI:** 10.1038/cddis.2016.390

**Published:** 2016-11-24

**Authors:** Dana Thomasova, Martrez Ebrahim, Kristina Fleckinger, Moying Li, Jakob Molnar, Bastian Popper, Helen Liapis, Ahmed M Kotb, Florian Siegerist, Nicole Endlich, Hans-Joachim Anders

**Affiliations:** 1 Nephrologisches Zentrum, Medizinische Klinik und Poliklinik IV, Klinikum der LMU München, Munich, Germany; 2 Department of Anatomy and Cell Biology, Ludwig-Maximilians Universität, Munich, Germany; 3 Pathology & Immunology & Internal Medicine (Renal), Washington University, School of Medicine, St Louis, MO, USA; 4 Department of Anatomy and Cell Biology, Universitätsmedizin Greifswald, Greifswald, Germany; 5 Department of Anatomy and Histology, Faculty of Veterinary Medicine, Assiut University, Assiut, Egypt

## Abstract

Murine double minute-2 (MDM2) is an E3-ubiquitin ligase and the main negative regulator of tumor suppressor gene p53. MDM2 has also a non-redundant function as a modulator of NF-kB signaling. As such it promotes proliferation and inflammation. MDM2 is highly expressed in the unchallenged tubular epithelial cells and we hypothesized that MDM2 is necessary for their survival and homeostasis. MDM2 knockdown by siRNA or by genetic depletion resulted in demise of tubular cells *in vitro*. This phenotype was completely rescued by concomitant knockdown of p53, thus suggesting p53 dependency. *In vivo* experiments in the zebrafish model demonstrated that the tubulus cells of the larvae undergo cell death after the knockdown of mdm2. Doxycycline-induced deletion of MDM2 in tubular cell-specific MDM2-knockout mice *Pax8rtTa-cre; MDM2f/f* caused acute kidney injury with increased plasma creatinine and blood urea nitrogen and sharp decline of glomerular filtration rate. Histological analysis showed massive swelling of renal tubular cells and later their loss and extensive tubular dilation, markedly in proximal tubules. Ultrastructural changes of tubular epithelial cells included swelling of the cytoplasm and mitochondria with the loss of cristae and their transformation in the vacuoles. The pathological phenotype of the tubular cell-specific MDM2-knockout mouse model was completely rescued by co-deletion of p53. Tubular epithelium compensates only partially for the cell loss caused by MDM2 depletion by proliferation of surviving tubular cells, with incomplete MDM2 deletion, but rather mesenchymal healing occurs. We conclude that MDM2 is a non-redundant survival factor for proximal tubular cells by protecting them from spontaneous p53 overexpression-related cell death.

Renal tubular epithelial cells are continuously exposed to stress due to the hypoxia, hyperosmolarity and toxins exposure and it is rather remarkable that they can withstand those insults and still execute their physiological functions that is, water and solutes reabsorption and excretion. Acute exposures can lead to acute tubular necrosis underlying the clinical syndrome of acute kidney injury. In unchallenged kidneys, tubular epithelial cells divide at a very low rate. This minimal production of new cells supplies though enough tubular cells to balance the loss of the tubular epithelial cells into urine and guarantees the physiological turnover of tubule cells. Nevertheless, this turnover rate must be strictly controlled as even a small disproportion between cell death and cell proliferation would eventually result in nephron loss or significant increase in nephron size.^1, 2^ In unstressed kidney remain the tubular cells in G0–G1, quiescent state.^3^ The mechanisms and factors necessary for the tubule cells homeostasis are not fully understood.

E3-ubiquitin ligase murine double minute-2 (MDM2) is the master negative regulator of tumor suppressor gene p53 and a non-redundant modulator of NF-ĸB signaling.^4, 5^ As such MDM2 amplification or overexpression drives tumor growth and MDM2 blockade suppresses cancer development.^6, 7^ In acute kidney injury caused by primary glomerular insults, MDM2 rather fosters podocyte demise by driving the podocytes into mitosis, pushing them to bypass the G2/M checkpoint that is, mitotic catastrophe.^8^ Moreover, by facilitating the NF-ĸB signaling, MDM2 promotes glomerular inflammation in injured glomeruli and thus further aggravates the podocyte loss, endothelial damage and glomerulosclerosis.^9^ In acute tubular injury MDM2 exacerbates the initial damage phase via NF-ĸB-related inflammation but promotes regeneration in the later healing phase via p53 regulation.^10^ In podocyte homeostasis MDM2 functions as a crucial factor protecting podocytes from p53 overactivation related cell death contributing thus to the lifelong survival of podocytes.^11^ Resting tubular epithelial cells express high levels of MDM2 and we hypothesized that quiescent tubular epithelial cells require MDM2 to maintain the homeostasis. To address this hypothesis we depleted the MDM2 or both MDM2 and p53 in cultured murine tubular epithelial cells and in primary tubule cells and in the mouse model by generating the tubular epithelial cells-specific knockouts.

## Results

### MDM2 prevents tubular epithelial cell death *in vitro*

**Figure 1:**
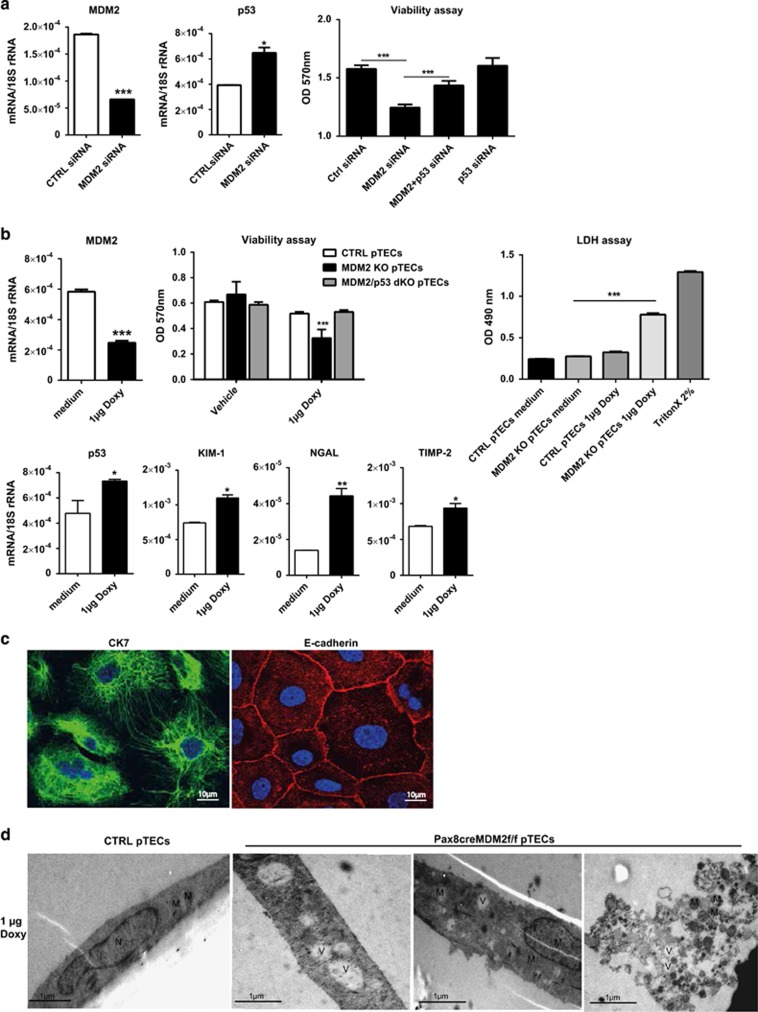
MDM2 knockdown/knockout in murine tubular cell line and in primary tubular cells. (**a**) Murine tubular epithelial cells were incubated for 24 h with MDM2 siRNA or control scrambled siRNA and MDM2 and p53 mRNA expression was measured by RT-PCR. The viability was assessed by MTT assay. (**b**) Primary tubular epithelial cells (pTEC) were isolated from *Pax8-rtTAcre;MDM2f/f* (MDM2KO pTECs) or *Pax8-rtTAcre;MDM2f/f;p53f/f* (MDM2/p53dKO pTECs) mice and treated with doxycycline to induce MDM2 or MDM2/p53 knockout. mRNA expression of MDM2, p53 and tubular damage markers KIM-1, NGAL and TIMP-2 was determined by RT-PCR. Cell viability and cell death were measured by MTT and LDH assays. CTRL pTECs were isolated from *MDM2fl/fl* mice. CTRL pTECs treated with 2% Triton X were used as positive control. (**c**) Representative confocal microscopy pictures of pTECs stained for markers of tubular epithelial cells cytokeratine-7 and E-cadherin used to assess the purity of the cell isolates. (**d**) Electron microscopy images of control and MDM2KO pTECs treated with doxycycline. The MDM2-depleted pTECs undergo cell death associated with prominent cytoplasmic vacuolization, cell membrane breaks and finally with the complete disassembly of the cell structure. All experiments were performed in triplicate. Data are means±S.E.M. * *P*<0.05, **P<0.01, ***P<0.005. N, nucleus; M, mitochondria; V, vacuole

The knockdown (KD) of MDM2 in mouse tubular epithelial cell line with siRNA resulted in decreased viability of tubular cells in comparison to tubular cells treated with control siRNA (Figure 1a). MDM2 suppression was associated with a marked increase in p53 and p53 effector genes p21 and PUMA transcript levels (Supplementary Figure 1A). The concomitant KD of MDM2 and p53 significantly improved the viability of tubular epithelial cells *in vitro* (Figure 1a). This result was confirmed in primary tubular cells MDM2 KO pTECs isolated from *Pax8rtTa-cre; MDM2f/f* mice, where MDM2 was depleted specifically in tubular epithelial cells *in vitro* by treatment with doxycycline. The generation of theses mice is described below. MDM2 mRNA levels decreased significantly in MDM2 KO pTECs treated with 1μg doxycycline (Figure 1b). The Mdm2-deficient primary tubular cells showed increased expression of tubular damage markers KIM-1, NGAL and TIMP-2 as well as increased cell death, due to the upregulation of p53 (Figure 1b). Increased p53 activity was confirmed by elevated mRNA expression of p53-target genes p21 and PUMA (Supplementary Figure 1B). The simultaneous depletion of MDM2 and p53 completely rescued the viability of the primary tubular cells (Figure 1b). The pTECs population was about 95% pure as assessed by staining for the tubular epithelial cell markers cytokeratin-7 and E-cadherin (Figure 1c). To prove the specificity of MDM2 depletion in tubular epithelial cells, we isolated also parietal epithelial and mesangial cells from kidneys of *Pax8rtTA-cre; MDM2f/f*, treated them with doxycycline, and checked mRNA levels of MDM2 and viability of those cells. Unlike in primary tubular cells, MDM2 expression and viability remained unchanged in the glomerular cells in comparison to the controls (Supplementary Figure 1C). Ultrastructural analysis of murine primary tubular cells lacking MDM2 revealed vacuolization of cytoplasm, nicks in the cytoplasmic membrane, and eventually cell death with complete disassembly of the cell structure (Figure 1d). These results suggest that MDM2 is required to protect the tubular cells from p53 overexpression-related cell death.

### Mdm2 KD in zebrafish larvae induces cell death of renal tubular epithelial cells

**Figure 2:**
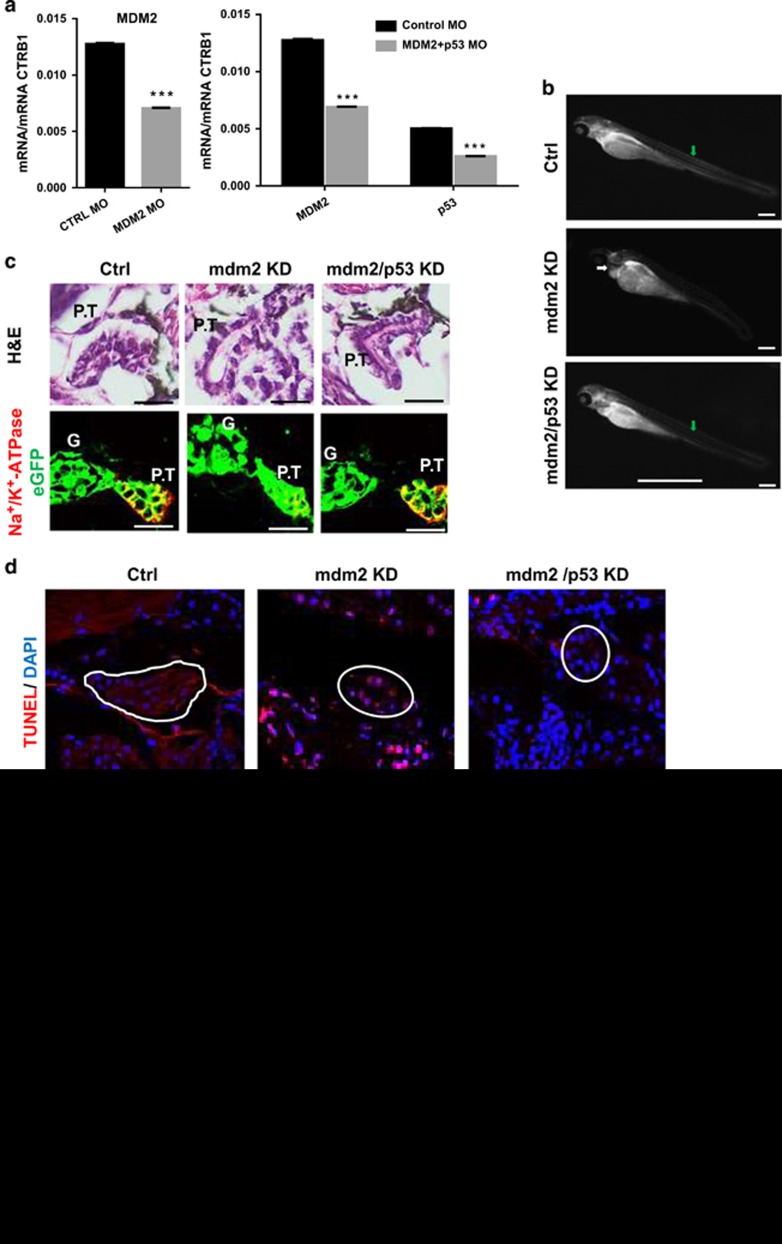
Mdm2 knockdown in zebrafish larvae. (**a**) After the treatment of fertilized zebrafish eggs with mdm2 MO, mdm2/p53MO, mRNA expressions of mdm2 or both mdm2 and p53 were significantly reduced in contrast to larvae treated with Ctrl MO. The target mRNA expression levels were determined by real-time RT-PCR and expressed as mean of the ratio *versus* the respective ctrb1 mRNA level±S.E.M. Data are means±S.E.M. ***P<0.005. (**b**) CADE larvae developed pericardial edema (white arrow) after the mdm2 KD in contrast to control morpholino-injected larvae (Ctrl) and mdm2/p53 morpholino-injected larvae. Mdm2 KD larvae showed a loss of the vascular eGFP fluorescence (green arrow) due to a leaky filtration barrier. Scale bar represents 50 *μ*m. (**c**) Paraffin sections were stained with H&E and an antibody against Na^+^/K^+^–ATPase, respectively. Glomerulus (G), proximal tubule (P.T). Scale bar represents 20 *μ*m. (**d**) TUNEL assay showed dead cells in the pronephric tubules of mdm2 KD larvae. Scale bar represents 20 *μ*m. (**e**) 3D reconstructions of 2-PM z-stack showed dilated pronephric proximal tubules in mdm2 KD larva. Scale bar represents 20 *μ*m. (**f**) The box plot histogram shows the significant dilatation of pronephros proximal tubule lumen of mdm2 KD larvae compared with Ctrl and mdm2/p53 KD larvae. Scale bar 20 *μ*m

To validate the *in vitro* results *in vivo*, a KD of mdm2 was generated in zebrafish larvae by the injection of specific morpholinos into fertilized eggs and was confirmed by qRT-PCR (Figure 2a). At 4 days post fertilization (d.p.f.), the mdm2 KD zebrafish larvae developed pericardial edema (white arrow in Figure 2b), a hallmark of kidney failure, in contrast to control morpholino-injected larvae (Ctrl) and larvae with a double KD knockout of mdm2 and p53 (mdm2/p53). For a rapid screening of the glomerular filtration barrier function, we used the transgenic zebrafish strain CADE^12^ that expresses the eGFP-labeled protein of the albumin family *gc* (group specific component) in the blood which is unable to pass the healthy filtration barrier. In Figure 2b, it is shown that the KD of mdm2 resulted in larvae with reduced eGFP fluorescence in the vessels indicating a leakage of the filtration barrier. To investigate the morphology of pronephric tubules after the KD of mdm2, we used the zebrafish strain Tg(*wt1b*:eGFP) that expresses eGFP in podocytes, parietal epithelial cells and proximal tubule cells.^13^ Sections of zebrafish larvae revealed a significant dilatation of the pronephric tubules in mdm2 KD larvae (Figure 2c) which could be rescued to some extent by the KD of p53. Additionally, we have found less epithelial cells in the proximal tubules after the mdm2 KD compared with Ctrl and mdm2/p53 KD larvae (Figure 2c). The TUNEL assay demonstrated that this is due to an increase of dead cells in mdm2 KD larvae (Figure 2d). Furthermore, the remaining cells showed a reduced expression of the Na^+^–K^+^–ATPase, a marker of differentiated tubular epithelial cells (Figure 2c).

*In vivo* imaging of the pronephros of living Tg(wt1b:eGFP) larvae by two-photon microscopy (2-PM) confirmed the significant dilatation of the Bowman space after mdm2 KD (asterisk in Figure 2e; Supplementary Movie 1) in contrast to mdm2/p53 and Ctrl KD larvae (Supplementary Movies 2 and 3). We have found that the maximum width of the pronephric proximal tubules in living zebrafish larvae significantly increased from 7.03 *μ*m (S.E.M.=2.29 *μ*m, *n*=7) in Ctrl and 8.53 *μ*m (S.E.M.=2.19 *μ*m, *n*=7, *P*=0.01) in mdm2/p53 KD larvae to 14.67  *μ*m (S.E.M.=2.42 *μ*m, *n*=9, *P*=0.044) in mdm2 KD larvae (Figure 2f). These results show that mdm2 is essential for pronephric tubular epithelial cells by protecting them from a p53-dependent cell death.

### MDM2 depletion in tubular epithelium results in acute kidney injury

**Figure 3:**
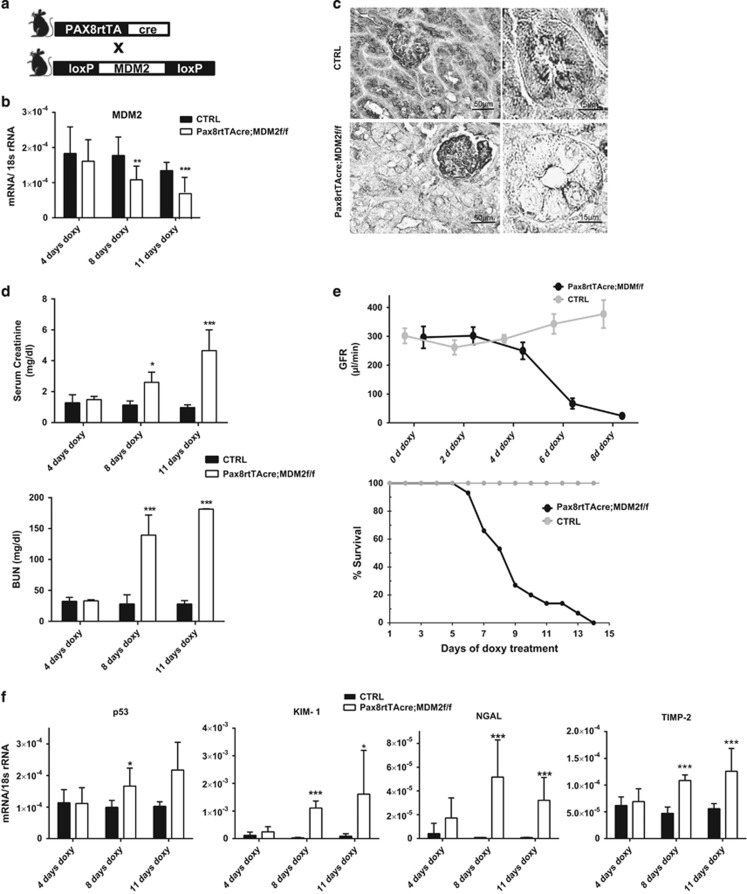
Tubular cell-specific MDM2 knockout in mouse model results in acute kidney injury with fast decline of renal function. (**a**) Mice expressing inducible cre recombinase under control of Pax8 promoter were crossed with MDM2^*fl/fl*^mice to generate tubular cell-specific MDM2 knockout mice. (**b**) RT-PCR analysis confirmed suppression of MDM2 mRNA expression in kidney lysates of *Pax8rtTA-cre;MDM2f/f* mice treated with doxycycline. (**c**) Immunohistochemical staining confirmed MDM2 deletion in tubular cells while in podocytes in glomerulus the MDM2 expression remained unchanged. (**d**) *Pax8rtTA-cre;MDM2f/f* mice treated with doxycycline developed increasing elevation of plasma creatinine and plasma BUN (*n*=5–6 mice in each group). (**e**) Left: GFR measurement showed significant decrease of GFR from day 6 of doxycycline treatment in *Pax8rtTA-cre;MDM2f/f* mice while the GFR of control MDM2f/f mice was unaffected. Right: Kaplan–Meyer survival curve of MDM2^−/−tubulus^ and control mice (*n*=17 per group). (**f**) mRNA expression of p53 as well as of tubules damage markers KIM-1, NGAL and TIMP-2 was increasingly elevated in MDM2^−/−tubulus^ mice kidneys in comparison to control mice kidneys. Data are means±S.E.M. *P<0.05, **P<0.01, ***P<0.005

**Figure 4:**
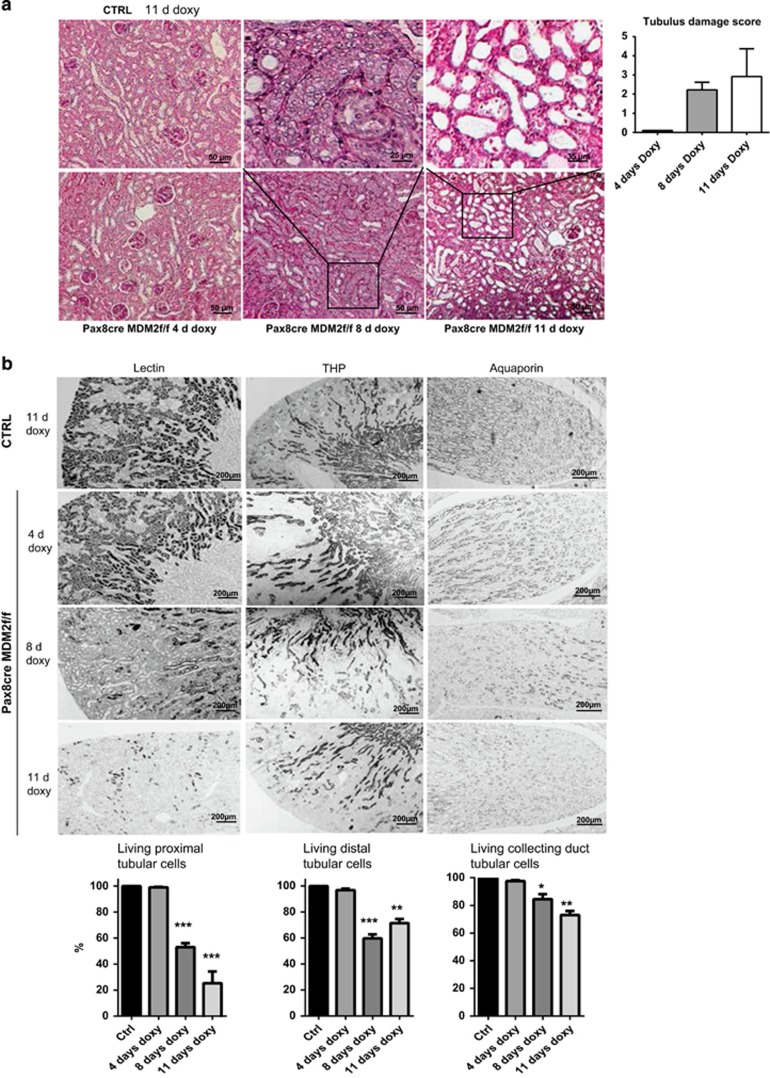
Tubular injury in tubular cell-specific MDM2 knockout mice kidneys. (**a**) Representative images of PAS-stained kidney sections at a magnification 200x from *Pax8rtTA-cre;MDM2f/f* and control mice treated for 4, 8 or 11 days with doxycycline showing the progressive aggravation of tubular injury. At day 4 was no tubular damage apparent, at day 8 the tubular cells swelling and vacuolization were the prominent pathologic features and at day 11 the global tubular damage with massive cell loss, tubular dilation and tubular casts was detected. Tubular injury was quantified on PAS-stained renal section as described in Materials and methods. (**b**) *Lotus tetragonolobus* lectin staining identified proximal tubuli, Tamm-Horsfall protein (THP) staining identified distal tubuli and Aquaporin 2 identified the collecting ducts in *Pax8rtTA-cre;MDM2f/f* and control mice kidneys treated for 4, 8 or 11 days with doxycycline. The quantitative assessment of tubuli with intact staining patterns is shown for each staining. Data are means±S.E.M. from six mice in each group. *P<0.05, **P<0.01, ***P<0.005. All images are shown at a magnification of × 100

**Figure 5:**
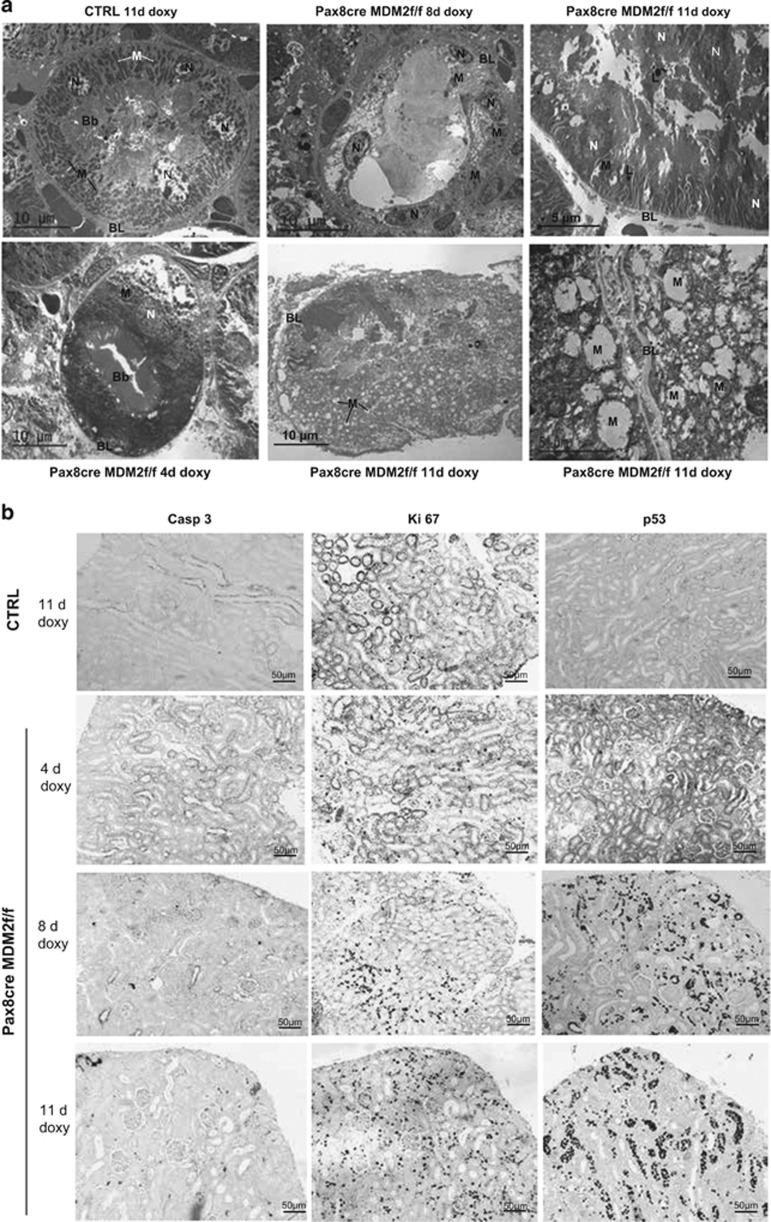
Ultrastructural pathology in tubular cell-specific MDM2 knockout mice tubular cells. (**a**) Electron microscopy at 4 days of MDM2 depletion reveled an onset of cytoplasmic swelling, at 8 days pronounced cytoplasmic swelling with occasional cytoplasmic membrane rupture but intact nuclei and at 11 days a complete degradation of the tubular cell structure with vacuolization of swollen cytoplasm, massive degeneration of mitochondria, edematous nuclei and release of the cell content into the tubular lumen. (EM in successive low–medium–high 1500–12 000 magnification). (**b**) Representative pictures of kidney sections from *Pax8rtTA-cre;MDM2f/f* and control mice kidneys treated for 4, 8 or 11 days with doxycycline showing staining for activated caspase-3, marker of apoptosis, Ki-67, marker of proliferation or hypertrophy and p53, marker of the cell cycle arrest, senescence and cell death. All images are shown at a magnification of × 100. Bb, brush border; BL, basal lamina; L, lysosome; M, mitochondria; N, nucleus

To confirm these findings in the mammalian kidney, we crossed Pax8rtTA-cre mice with *MDM2f/f* mice to generate a mice model with inducible MDM2 deletion exclusively in renal tubular epithelial cell upon doxycycline treatment, *Pax8rtTA-cre;MDM2f/f* (Figure 3a). These mice were born at expected Mendelian ratios and did not show any functional or renal histological abnormalities within 6 months of age. Real-time PCR analysis of kidney extracts from *Pax8rtTA-cre;MDM2f/f* mice treated with doxycycline (MDM2^−/−tubulus)^ for 4, 8 or 11 days demonstrated progressively declining levels of MDM2 mRNA compared with control mice (Figure 3b). MDM2 immunostaining was selectively diminished in the renal tubular cells of the *Pax8rtTA-cre;MDM2f/f* mice treated with doxycycline (MDM2^−/−tubulus^) while the MDM2 immunostaining remained intact in the glomerulus, confirming thus the selectivity of the MDM2 tubular deletion (Figure 3c; Supplementary Figure 2A). MDM2^−/−tubulus^ mice showed progressive impairment of kidney function in comparison to control mice, as documented by significant increase of plasma creatinine and blood urea nitrogen (BUN) levels (Figure 3d). Measured glomerular filtration rate (GFR) declined from day 4 of doxycycline treatment in MDM2^−/−tubulus^ mice and at day 8 the mice were severely oliguric compared with control littermates (Figure 3e). The decline of MDM2^−/−tubulus^ mice kidney function was also associated with the shortened lifespan (Figure 3e). Also the renal mRNA levels of p53 and its effector genes p21 and PUMA and tubular damage markers KIM-1, NGAL and TIMP-2 had increased with the length of doxycycline treatment (Figure 3f; Supplementary Figure 2B). On light microscopy the kidneys of MDM2^−/−tubulus^ mice appeared normal at 4 days of doxycycline treatment while on day 8 profound tubular epithelial cells swelling and their vacuolization were apparent (Figure 4a). At day 11 of the doxy treatment the kidneys displayed massive tubular dilation with tubular cell flattening, damage and cell loss and multifocal tubular granular casts (Figure 4a). Although the MDM2 deletion in tubular epithelial cells affected both proximal and distal tubuli as well as collecting ducts, the proximal tubuli exhibited the highest damage with the loss of almost 80% of the proximal tubular cells as determined by staining for *Lotus tetragonolobus* lectin (Figure 4b). Analysis of the kidneys of MDM2^−/−tubulus^ mice by electron microscopy detected ultrastructural abnormalities in tubular epithelial cells such as swelling of the cytoplasm, endoplasmic reticulum changes, swelling of the mitochondria with the loss of cristae and their transformation in the vacuoles (Figure 5a). The nuclei were edematous but with no membrane rupture and they did not appear to have consolidated chromatin (Figure 5a). These abnormalities suggest that the tubular epithelial cells undergo a stage of asphyxia and start dying from the cytoplasm with dead mitochondria. Eventually, the tubular epithelial cells completely disintegrate, the plasma membrane ruptures, and the nuclei are released together with other cytoplasmic organelles (Figure 5a). Lack of MDM2 in tubular epithelial cells was associated with the progressive increase of p53 expression and its nuclear shift. Simultaneously we observed also the Ki-67 activation, suggesting the hypertrophy or proliferation of undamaged tubular epithelial cells with incomplete MDM2 deletion or of the tubular progenitors (Supplementary Figure 3A). Nevertheless, the regenerating tubular cells were not able to compensate for the cellular loss due to p53 overactivation. TUNEL as well as cleaved caspase-3 staining showed just marginal caspase-3 activation in the tubular compartment, mostly in the kidneys with marked p53 upregulation and massive tubular cell death (Figure 5b; Supplementary Figure 3B). The caspase-3 activation level does not correspond to the extent of the kidney damage and is suggestive of secondary cell apoptosis. We conclude that MDM2 is essential for tubular epithelial cells homeostasis and survival and prevents acute kidney injury.

### The MDM2 deleterious phenotype in tubular epithelium is p53 dependent

**Figure 6:**
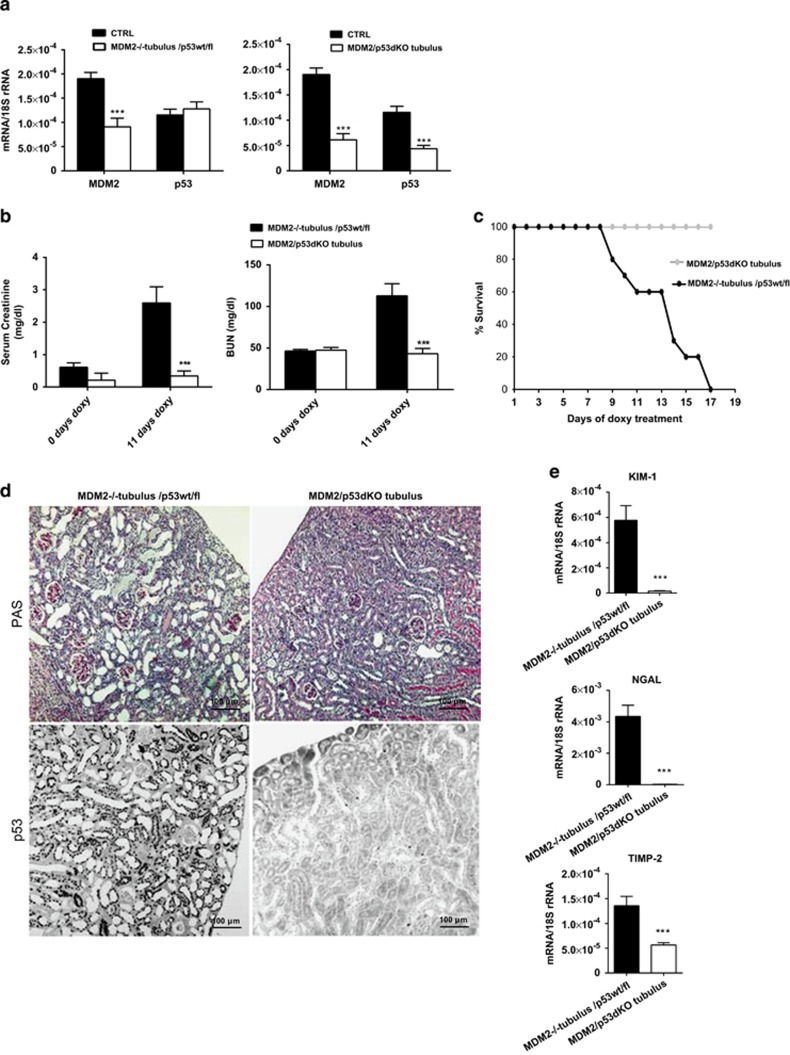
Simultaneous MDM2 and p53 tubular cell-specific knockout rescues the pathological phenotype in MDM2/p53^dKO tubulus^ mouse model. (**a**) Total mRNA was prepared from kidneys isolated from MDM2^−/−tubulus^ /p53^*wt/fl*^*or* MDM2/p53^dKO tubulus^ mice respectively and control MDM2^*fl/fl*^mice treated for 11 days with doxycyclin. The target mRNA expression levels were determined by real-time PCR and expressed as mean of the ratio *versus* the respective 18S mRNA level±S.E.M. (**b**) Serum creatinine and BUN measurement in plasma of MDM2/p53^dKO tubulus^ and positive control MDM2^−/−tubulus^ /p53^*wt/fl*^mice treated for 11 days by doxycycline (*n*=10 in each group). (**c**) Kaplan–Meyer survival curve of MDM2/p53^dKO tubulus^ and control MDM2^−/−tubulus^ /p53^*wt/fl*^mice (*n*=10 in each group). (**d**) Representative images of PAS and p53 stained kidney sections from MDM2/p53^dKO tubulus^ and control MDM2^−/−tubulus^ /p53^*wt/fl*^mice treated for 11 days with doxycycline. All images are shown at a magnification of × 200. (**e**) Real-time PCR analysis of renal tubular damage markers KIM-1, NGAL and TIMP-2 mRNA prepared from kidneys isolated from MDM2/p53^dKO tubulus^ and positive control MDM2^−/−tubulus^ /p53^*wt/fl*^mice treated for 11 days with doxycycline. Data are means±S.E.M. ***P<0.005

To test for the role of p53 in the structural and functional renal pathology of MDM2^−/−tubulus^ mice, we generated inducible tubular epithelial cell-specific simultaneous MDM2 and p53 knockout mice MDM2/p53^dKO tubulus^ and the littermate controls with one p53 allele intact MDM2^−/−tubulus^ /p53^*wt/fl*^. We treated the both mouse lines with doxycycline to induce the deletion of the respective gene alleles. The analysis of the functional and histological parameters of the mice models confirmed that the depletion of p53 rescues the pathological phenotype of the MDM2^−/−tubulus^ mice (Figures 6a–e).

### Tubular epithelium compensates only partially for the cell loss caused by MDM2 depletion

**Figure 7:**
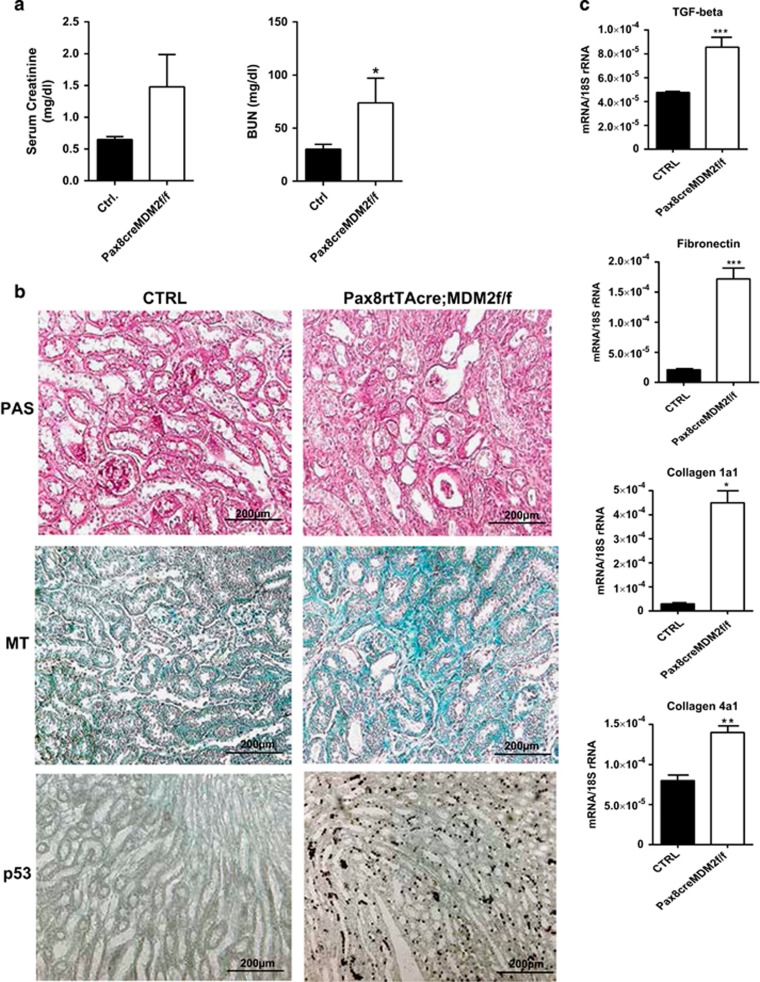
Tubular epithelium compensates only partially for the cell loss caused by MDM2 depletion. To induce a sub-lethal deletion of MDM2 in tubular epithelial cells in our mouse kidney model we treated *Pax8rtTA-cre;MDM2f/f* mice intermittently with 2 mg/ml doxycycline for 4 weeks. (**a**) *Pax8rtTA-cre;MDM2f/f* mice treated with intermittent regimen of doxycycline had elevated plasma creatinine and plasma BUN at 4 weeks (*n*=5–6 mice in each group). (**b**) Representative images of kidney sections from control *MDM2f/f* and *Pax8rtTA-cre;MDM2f/f* mice treated for 4 weeks intermittently with doxycycline and stained with PAS, Masson trichrome and p53 antibody. All images are shown at a magnification of × 200. (**c**) Real-time PCR analysis of pro-fibrotic genes mRNA prepared from kidneys isolated from experimental and control mice treated for 4 weeks with doxycycline. Data are means±S.E.M. *P<0.05, **P<0.01, ***P<0.005

Previously, it was reported that mice lacking Mdm2 in the intestinal epithelium can fully compensate for the MDM2 depletion/p53 activation-mediated cellular loss because of negative selection of the MDM2 transgenic cells and rapid proliferation and overgrowth of cells with insufficient Cre recombinase activity and thus incomplete MDM2 depletion.^14^ To investigate whether this phenomenon occurs also in renal tubular epithelium, which is known for its fast regenerative capacity, we subjected the *Pax8rtTA-cre; MDM2f/f* mice to different doxycycline treatment regimens to avoid the rapid lethality of the continuous doxycycline treatment. We treated the mice with doxycycline for 2 days to deplete the MDM2 just in the portion of the tubular epithelial cells and repeated the treatment after 5 days of recovery for three times. The renal function of the experimental mice was impaired compared with the control mice as documented by elevated serum creatinine and BUN levels (Figure 7a) but the lifespan of the experimental animals was not affected (data not shown). The histological analysis showed focal tubular damage with overproduction of extracellular matrix, suggesting that the loss of tubular cells is only partially recovered by surviving tubular cells but rather mesenchymal repair occurs (Figure 7b). Masson trichrome staining confirmed the accumulation of the fibrotic tissue in the kidneys of experimental mice (Figure 7b) and also mRNA expression levels of several pro-fibrotic genes were elevated (Figure 7c). We detected moderate p53 activation with nuclear shift throughout the tubular compartment with especially high expression in medullar region of the kidney (Figure 7b). We conclude that unlike intestinal epithelium, tubular epithelial cells cannot completely compensate for the cells lost due to the MDM2 deletion.

## Discussion

We hypothesized that MDM2 is important for homeostasis of resting tubular epithelial cells in kidney. In the cell culture experiments the tubular epithelial cells lacking MDM2 showed decreased viability. The mouse model with the specific deletion of MDM2 in tubular epithelial cells displayed shortened lifespan, fast decline in renal function as documented by reduced GFR and increased plasma creatinine and BUN. We also detected significant upregulation of tubular damage markers and massive swelling, mitochondrial degeneration and cell death in the tubular compartment in the kidneys of the experimental animals. All these changes developed in unchallenged kidney and were consistent with acute tubular injury. The tubular epithelium was not able to fully compensate for the cell loss due to the MDM2 depletion by proliferation of remaining tubular cells with intact MDM2 but rather mesenchymal healing with fibrosis occurred. The pathological phenotype was completely rescued by co-deletion of p53. We conclude that MDM2 is essential for survival and homeostasis of intact tubular cells in healthy kidney.

MDM2, the main negative regulator of p53 suppresses its function in three different ways; it chaperons p53 out of the nucleus, that is, out of the transcriptional centrum, it blocks the transcription of p53 effector genes and it ubiquitinates p53 and targets it for proteasomal degradation.^4^ Through the p53 inhibition, MDM2 fosters cell proliferation and prevents p53-mediated cell death, cell cycle arrest and senescence.^15^ This MDM2 function is indispensable for the embryonic development, wound healing and regeneration^16^ but it also facilitates autoimmune disorders, such as lupus erythematosus^17^ and carcinogenesis.^18, 19^ MDM2 is overexpressed frequently in many human tumors, especially in breast cancer and sarcomas.^20^ The inhibitors of MDM2 and p53 binding are being currently tested in clinical trials as potential cancer therapy.^21, 22^ Nevertheless also MDM2 depletion has deleterious effects as documented by embryonic lethality of a mouse model with genetic-deletion of MDM2. The mouse embryos die due to the uncontrolled p53-dependent cell death, while p53 deficiency completely rescues this deleterious phenotype.^23, 24^ Specific deletion of MDM2 in developing kidney results in impairment of renal progenitor cell expansion, in aberrant nephrogenesis and differentiation and leads to hypodysplasia of the kidneys.^25, 26, 27^ Our data are in line with these studies and show that MDM2 is crucial for the survival and homeostasis of unchallenged tubular epithelial cells in kidney. MDM2 prevents p53-mediated cell death of these renal cells. Our findings differ from a study which showed that unbuffered p53 activity caused by MDM2 deletion is detrimental only in radiosensitive tissues due to the massive cell loss, while the radio-insensitive tissues, including kidney, are protected from cell death but exhibit inhibition of cell proliferation.^28^ Our data indicate that MDM2 depletion in tubular epithelial cells leads to massive cell death due to p53 overactivation but proliferation activity is maintained as documented by increased expression of Ki-67, marker of proliferation. Our results are corroborated by another study showing that global MDM2 deletion results in morphological and functional abnormalities in both radiosensitive and radio-insensitive tissues, including kidney.^14^ Renal phenotype included initially just mild damage with no aberrant functional effect, but subsequently the kidneys exhibited severe injury with impairment of renal function.^14^ Further on, MDM2 deletion in intestinal epithelial cells results in multiple intestinal abnormalities in newborn animals.^29^ These abnormalities completely disappeared in adulthood due to the selection against the enterocytes lacking MDM2 and increased proliferation of the epithelium with intact MDM2.^29^ Although the renal tubular epithelium has an extensive regenerative capacity, our findings indicate that in kidneys of tubular specific depleted MDM2 mice the complete epithelial healing does not occur and is compensated by mesenchymal healing with extensive fibrosis. This suggests that the inhibition of MDM2 could lead to acute kidney injury and due to incomplete healing contribute to development of chronic kidney disease.^30^ In our previous studies we showed that MDM2 in acute kidney injury promotes tubular injury in early postischemic phase via augmentation of NF-kB signaling and thus inflammation in p53-independent manner.^10^ Our present data document, that all renal anomalies due to MDM2 depletion in unchallenged tubular epithelial cells are dependent on p53 activity, as the defects completely disappeared when p53 was absent. This is in line with studies which showed dose-dependent attenuation of tubular cell death and kidney injury in mouse model of cisplatin induced nephrotoxicity treated with p53 siRNA or with chemical inhibitor of p53.^31, 32^

Together, MDM2 is crucial in intact tubular epithelial cells in kidney to prevent spontaneous p53 overactivation dependent cell death and thus prevent acute tubular injury. As MDM2 antagonists are being developed as an alternative treatment to chemotherapy for cancer treatment, it is of note that MDM2 inhibition might be detrimental for normal tissues, especially for kidney as our data suggest. We conclude that MDM2 is essential for survival and homeostasis of tubular epithelium in kidney.

## Materials and Methods

### Cell culture experiments

Murine tubular epithelial cells (mTECs cell line) were maintained in DMEM medium supplemented with 10% fetal bovine serum and 1% penicillin/streptomycin.The primary tubular cells (pTECs) were prepared from renal cell suspensions as previously described.^33^ In brief, 4 weeks old Pax8-rtTAcre;MDM2f/f or MDM2f/f control mice were used for renal cell extraction. Decapsulated kidneys were diced and digested in 1 mg/ml collagenase A (Roche Diagnostics, Mannheim, Germany) for 30 min at 37 °C and then passed through 70 *μ*m pore sieve (BD, Franklin Lakes, NJ, USA), washed, and diluted in 2 ml of PBS. Separation of the tubular segments was achieved through Percoll (31%) centrifugation at 3000 r.p.m./10 min/4 °C. The pellet of tubular segments was collected and washed twice with PBS at 1500 r.p.m./5 min/4 °C. The renal tubular cell isolates were cultured under sterile conditions at 37 °C and 5% CO_2_ in conditioned medium consisting of DMEM w/glucose (Gibco/Life Technologies, Grand Island, NY, USA), 10% fetal bovine serum (Biochrom, Berlin, Germany), 1% penicillin/streptomycin (PAA Laboratories, Pasching, Austria), HBSS (Sigma-Aldrich, Steinheim, Germany), HEPES (Gibco/Life Technologies, Grand Island, NY, USA), EGF, T3, hydrocortisone, PGE-1 and insulin transferrin sodium selenite supplement (Roche Diagnostics, Mannheim, Germany). For the assessment of the purity of the pTECs population were the cells grown in the 8-well chamber slides to confluency, than fixed in ice-cold acetone, stained for the markers of the tubular epithelial cells cytokeratin-7 and E-cadherin and TOPRO for nuclei and analyzed with confocal microscopy. For the viability assays, the mouse mTECs or primary TECs were seeded in 96 well plates (8000 cells/well), for the RNA extraction the mTECs and pTECs were cultured in 6-well plates (200 000 cells/wellThe mTECs were then incubated with 25nM siRNA for 24–48 h using Lipofectamine RNAiMAX transfection reagent (Life Technologies, Darmstadt, Germany) for transient transfection. Specific siRNA to silence selectively MDM2 or p53 as well as appropriate control siRNA (Negative control No.1) was purchased from Ambion (Life Technologies). The pTECs isolated from *Pax8-rtTAcre;MDM2f/f* or *MDM2f/f* control mice were treated for 48 h with 1μg/ml doxycycline to induce MDM2 depletion. The TECs or the pTECs were collected, 24–48 h post-treatment for RNA isolation or for cytotoxicity/viability assays that were performed using LDH assay (Cytotoxicity Detection Kit (LDH), Roche, Mannheim, Germany) and MTT assay (CellTiter 96 Non-Radioactive Cell Proliferation Assay, Promega, Madison, WI, USA) according to manufacturer's instructions. Experiments were performed in triplicate.

### Zebrafish experiments:

Zebrafish strains and larvae were maintained as described previously.^34^ The following zebrafish strains were used: CADE (Tg (−3.5 fabp10a:gc-eGFP) *mitfa*^*w2/w2*^*; roy*^*a9/a9*^) which expresses a gc-eGFP fusion protein in the blood plasma.^12^ The (Tg(*wt1b*:eGFP)) strain that expresses eGFP under control of the wt1b promotor.^13^ The following morpholinos were used: mdm2 MO: 5′-AACAACTCTCTGTTGCCATTTTGGT-3′ p53 MO: 5′-GCGCCATTGCTTTGCAAGAATTG-3′ Crtl MO: 5′-CCTCTTACCTCAGTTACAATTTATA-3′. MOs were diluted to a concentration of 0.3 nM. A volume of ~3 nl per zebrafish egg was injected into the yolk at the one- to four-cell stage using a microinjector (Transjector 5246; Eppendorf, Germany). For histological staining, we followed our previously published protocols.^12^ Anti-A6f antibody (1:25, Sigma-Aldrich) was used.

2-PM experiments: To prevent pigmentation, the zebrafish larvae were subjected to E3 medium supplemented with 0.003% phenylthiourea at 1 d.p.f. For *in vivo* imaging experiments, zebrafish larvae were embedded in 0.8% low-melting agarose (Biozym LE agarose, Germany) in E3 medium in a dorsal side up position. After hardening the larvae were covered with E3 medium supplemented with 0.06 mg/ml Tricaine (Sigma-Aldrich, St Louis, MO, USA). All 2-PM experiments were performed at 22 °C. Z-stacks of pronephric glomeruli and proximal tubules were recorded over a distance of 98 *μ*m with a voxel volume of 0.30 × 0.30 × 2 *μ*m and reconstructed as 3D movies using the ZEN 2010 software (Carl Zeiss Microimaging, Jena, Germany) as described before.^35^ For significance testing Gaussian distribution was checked by Kolmogorov-Smirnov testing, followed by significance testing with Student's *t*-test using SPSS V.22 (IBM, Armonk, NY, USA). *P*-values below 0.05 were declared as statistical significant. All experiments were performed in accordance with German animal protection law overseen by the agencies of the Federal State of Mecklenburg - Western Pomerania.

### Mouse experiments

The Pax8-rtTAcre;MDM2f/f mice were generated by breeding the MDM2^*flox/flox*^ mice, in which loxP sites flanked the exon 4 and 5 of MDM2 gene, with Pax8-rtTAcre mice, expressing the inducible Cre recombinase under control of the Pax8 promoter. The MDM2^*flox/flox*^ mice (/B6 mixed background) were a generous gift from G. Lozano (University of Texas, Huston, USA) and the Pax8-rtTA mice (*C56Bl/6* background) were kindly provided by T. Huber (University of Freiburg, Freiburg, Germany). Both mice strains were previously described.^36, 37^ The MDM2^*flox/flox*^ littermates lacking the Pax8-rtTAcre transgene were used as control mice. To generate the tubular epithelial cell-specific MDM2/p53 double knockout *Pax8-rtTAcre;MDM2f/f;p53f/f* (MDM2/p53dKD tubulus) mice, we bred the *Pax8-rtTAcre;MDM2f/f* mice with p53^*flox/flox*^ mice, in which the loxP sites were inserted in intron 1 and 10 to ensure Cre-mediated removal of nearly all coding sequences.^38^ The p53^*flox/flox*^ mice were a generous gift from L. Rudolph (University of Ulm, Ulm, Germany). The *Pax8-rtTAcre;MDM2f/f;p53wt/f* (MDM2^−/−^tubulus/p53wt/fl) littermates were used as positive controls. To induce Cre recombinase expression, we treated the mice with 2mg/ml doxycycline in drinking water supplemented with 5% sucrose for various number of days as indicated in figures. All animal studies were approved by the Committee on Research Animal Care, Regierungspräsidium Oberbayern.

#### Renal function measurement

Plasma creatinine levels as well as BUN (DiaSys GmBH, Holzheim, Germany) levels were determined using commercially available kits as per manufacturer's protocol.

#### GFR measurement

GFR was measured transcutaneously using a USB-device according to manufacturer's instructions (Mannheim Pharma&Diagnostics GmbH, Mannheim, Germany). The USB-device was fasten with adhesive tape to the shaved area on the mouse back. The mice were injected with FITC-Sinistrin i.v. (15 mg FITC-Sinistrin/100 g body weight). The measurement was performed for 60 min and analyzed with software provided by the manufacturer.

#### Renal histology, immunohistochemistry, confocal microscopy and electron microscopy

Kidney tissues were fixed in 4% neutral-buffered formalin, dehydrated in graded alcohols and embedded in paraffin. For routine histology the 4 *μ*m sections were stained with periodic acid-Schiff (PAS) reagent. For immunohistochemistry sections were deparaffinized, rehydrated, transferred into citrate buffer, and either autoclaved or microwave treated for antigen retrieval and processed as described.^10^ For histochemistry we used biotinylated Lotus Tetragonolobus Lectin stain (Vector Labs, Burlingame, CA, USA), Tamm-Horsfall protein stain (Santa Cruz, CA, USA) and rabbit anti-mouse Aquaporin 2 (Abcam, Cambridge, UK). Further on the following primary antibodies were used: rabbit anti-mouse MDM2 (Abcam, Cambridge, UK), rabbit anti-mouse p53 (Vector Laboratories, Burlingame, CA, USA), rat anti-mouse Ki-67 (DakoCytomation, Glostrup, Denmark), rabbit anti-mouse cleaved caspase-3 (Cell signaling Technology, Denvers, MA, USA), mouse anti-mouse E-cadherin (BD Biosciences, San Jose, CA, USA) and mouse anti-mouse cytokeratin-7 (Abcam). Immunofluorescent stainings were evaluated using a LSM 510 confocal microscope and LSM software (Carl Zeiss AG). For transmition electron microscopy, the kidney cortex was sectioned into 1x1mm cubes and immediately immersed in fixative containing 3% glutaraldehyde and 1% paraformaldehyde in PBS. Post-fixation kidneys were immersed in cold fixative containing 2% glutaraldehyde and 2% paraformaldehyde in sodium cacodylate buffer (pH 7.4). Kidneys were postfixed in phosphate cacodylate-buffered 2% OhsO4 for 1 h, dehydrated in graded ethanols with a final dehydration in propylene oxide and embedded in Embed-812 (Electron Microscopy Sciences, Hatfield, PA, USA). Ultrathin sections (~90-nm thick) were stained with uranyl acetate and Venable's lead citrate and viewed with a JEOL model 1200EX electron microscope (JEOL, Tokyo, Japan).

#### Morphometry

To assess tubulointerstitial changes a semiquantitative score was established to evaluate the degree and extent of tubulointerstitial damage of each field and was graded from 0 to 4 as follows: 0 represents no lesion, 1 represents tubulointerstitial damage of less than 25% per field, and 2, 3 and 4 represent tubulointerstitial damage of 25–50%, 50–75% and more than 75% of the tubulointerstitium, respectively. Approximately 30 cortical and medullary visual fields (20 × ) per kidney were evaluated. Tubulointerstitial injury was defined by features such as tubular collapse, cast formation with tubular dilatation or atrophy, detachment of cells from the basement membrane.

### RNA preparation and real-time quantitative PCR

Total RNA was extracted from mouse glomeruli, isolated using Dynabead perfusion, or murine podocyte cell line using Ambion RNA extraction kit (Invitrogen, Carlsbad, CA, USA) following the manufacturer's instructions. After quantification, RNA quality was assessed using agarose gels before reverse transcriptionwith Superscript II (Invitrogen) as described.^39^ Real-time RT-PCR was performed using SYBRGreen PCR master mix and analyzed with a Light Cycler 480 (Roche). All gene expression values were normalized using 18s RNA as a housekeeping gene. All primers used for amplification were from Metabion (Martinsried, Germany). Primers used are listed in the Table 1.

### Statistical analysis

Values are expressed as mean±S.E.M. Statistical analysis was performed using Graphpad Prism5 software (GraphPad Software, La Jolla, CA, USA). Significance of differences was determined by the appropriate two-sided *t*-test for single comparisons. Analysis of variance (ANOVA) with *post hoc* Bonferroni's correction was used for multiple comparisons. *P* -values <0.05 were considered statistically significant.

## Figures and Tables

**Table 1:** Primers used for real-time RT-PCR

**Target**	**Primer sequence**	
MDM2	Forward primer	5′-TGTGAAGGAGCACAGGAAAA -3′
	Reverse primer	5′-TCCTTCAGATCACTCCCACC -3′
p53	Forward primer	5′-TCCGACTGTGACTCCTCCAT-3′
	Reverse primer	5′-CTAGCATTCAGGCCCTCATC-3′
KIM-1	Forward primer	5′-TGGTTGCCTTCCGTGTCTCT-3′
	Reverse primer	5′-TCAGCTCGGGAATGCACAA-3′
NGAL	Forward primer	5′-TGAACTTCTGAAAACGGCT-3′
	Reverse primer	5′-AGCAGCAAGGGCACAAT-3′
TIMP-2	Forward primer	5′-CGTTTTGCAATGCAGACGTA-3′
	Reverse primer	5′-GAATCCTCTTGATGGGGTTG-3′
TGF-β	Forward primer	5′-GGAGAGCCCTGGATACCAAC-3′
	Reverse primer	5′-CAACCCAGGTCCTTCCTAAA-3′
Fibronectin	Forward primer	5′-GGAGTGGCACTGTCAACCTC-3′
	Reverse primer	5′-ACTGGATGGGGTGGGAAT-3′
Collagen1a1	Forward primer	5′-ACATGTTCAGCTTTGTGGACC-3′
	Reverse primer	5′-TAGGCCATTGTGTATGCAGC-3′
Collagen4a1	Forward primer	5′-GTCTGGCTTCTGCTGCTCTT-3′
	Reverse primer	5′-CACATTTTCCACAGCCAGAG-3′
18s	Forward primer	5′-GCAATTATTCCCCATGAACG-3′
	Reverse primer	5′-AGGGCCTCACTAAACCATCC- 3′
