# Supplementary figures and images for: MDM2 prevents spontaneous tubular epithelial cell death and acute kidney injury
Source: Cell Death Dis. 2016 Nov 24;7(11):e2482–. doi: 10.1038/cddis.2016.390 (PMC5260907; doi:10.1038/cddis.2016.390)

Suppl. figure 1

A.

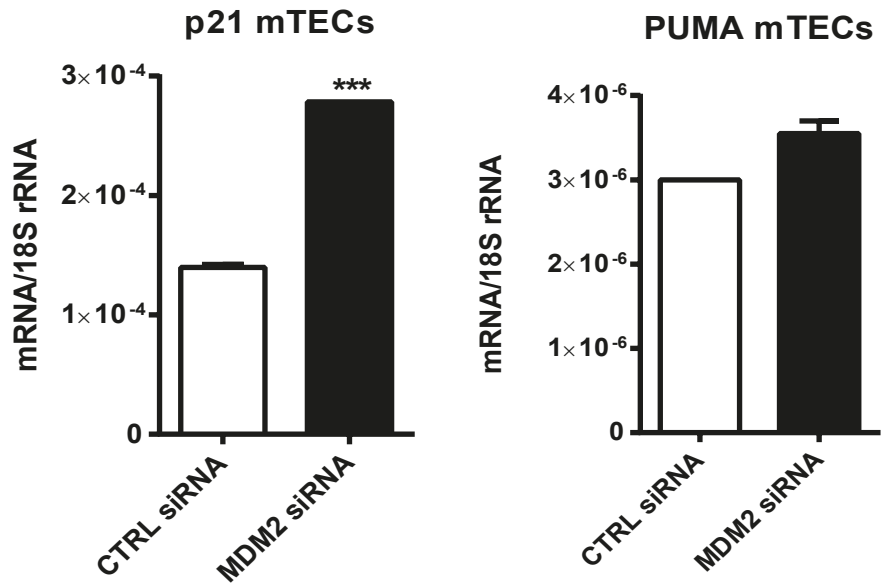

B.

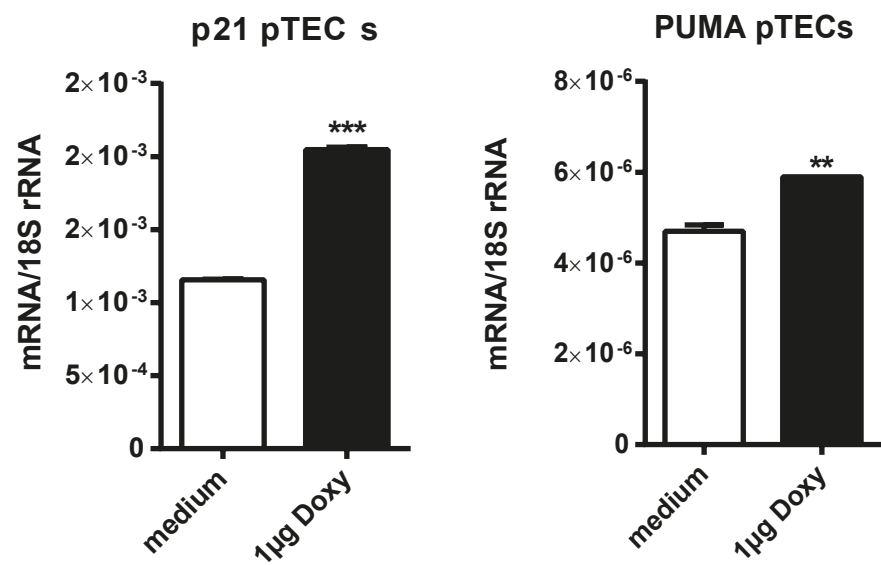

C.

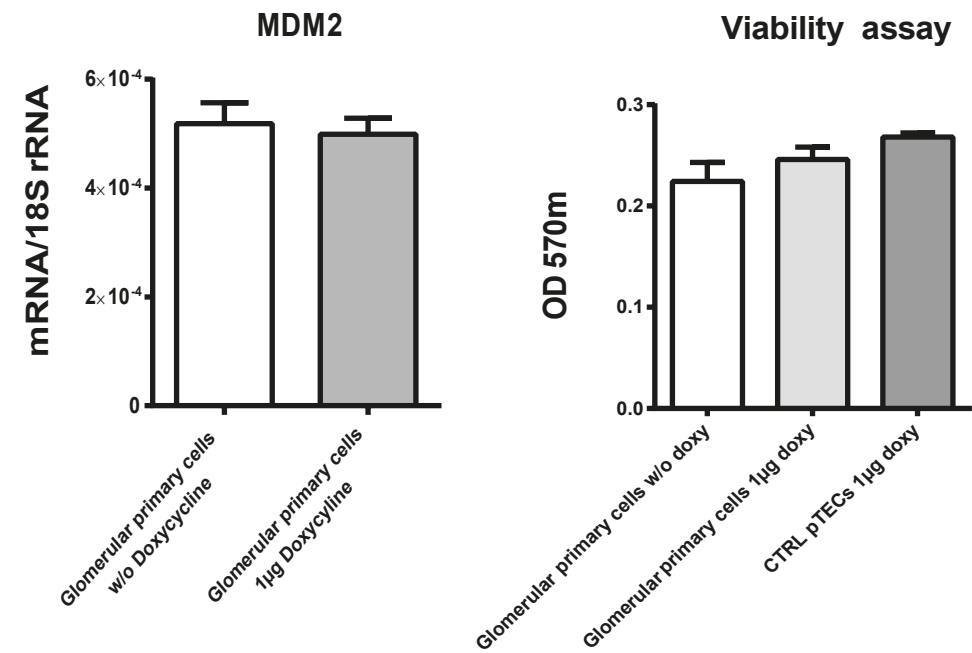

Suppl. Fig. 2

A.

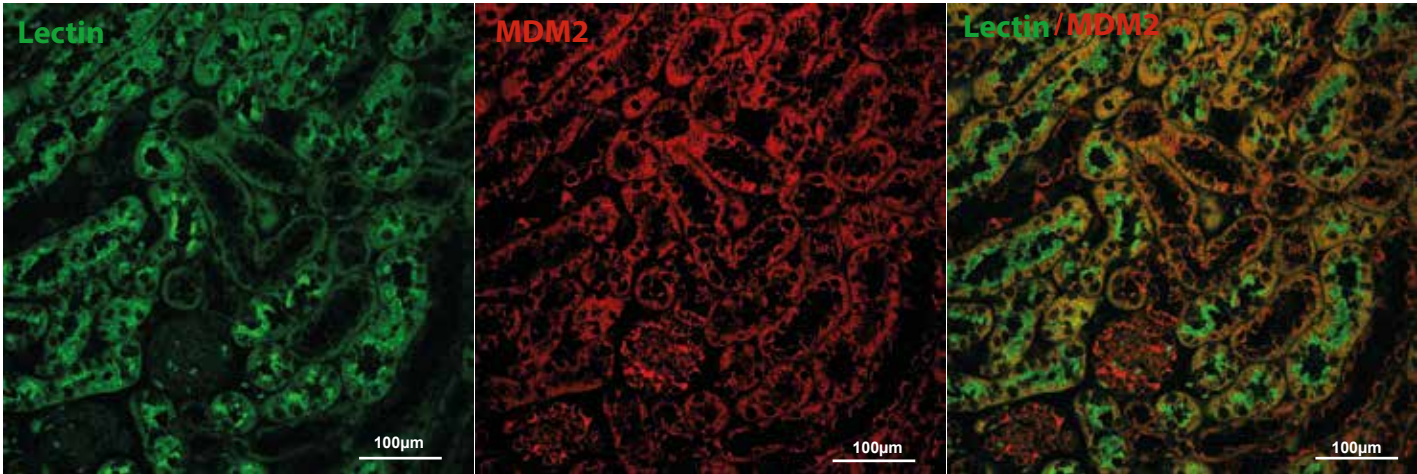

B.

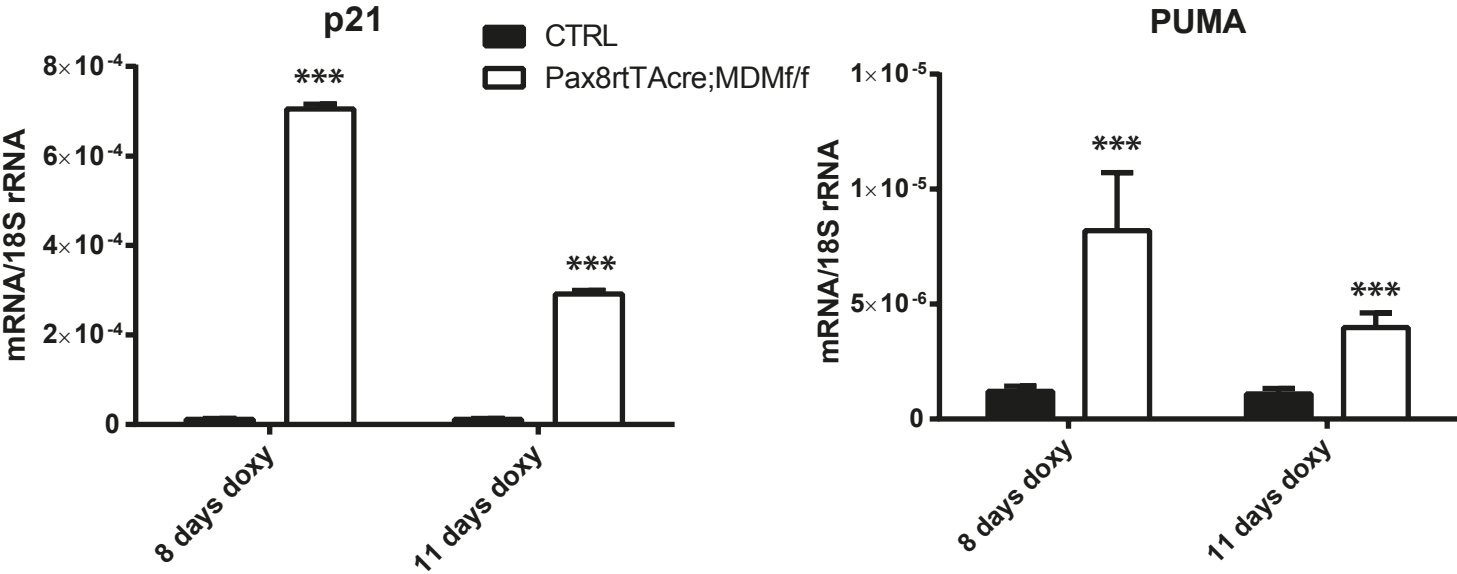

A

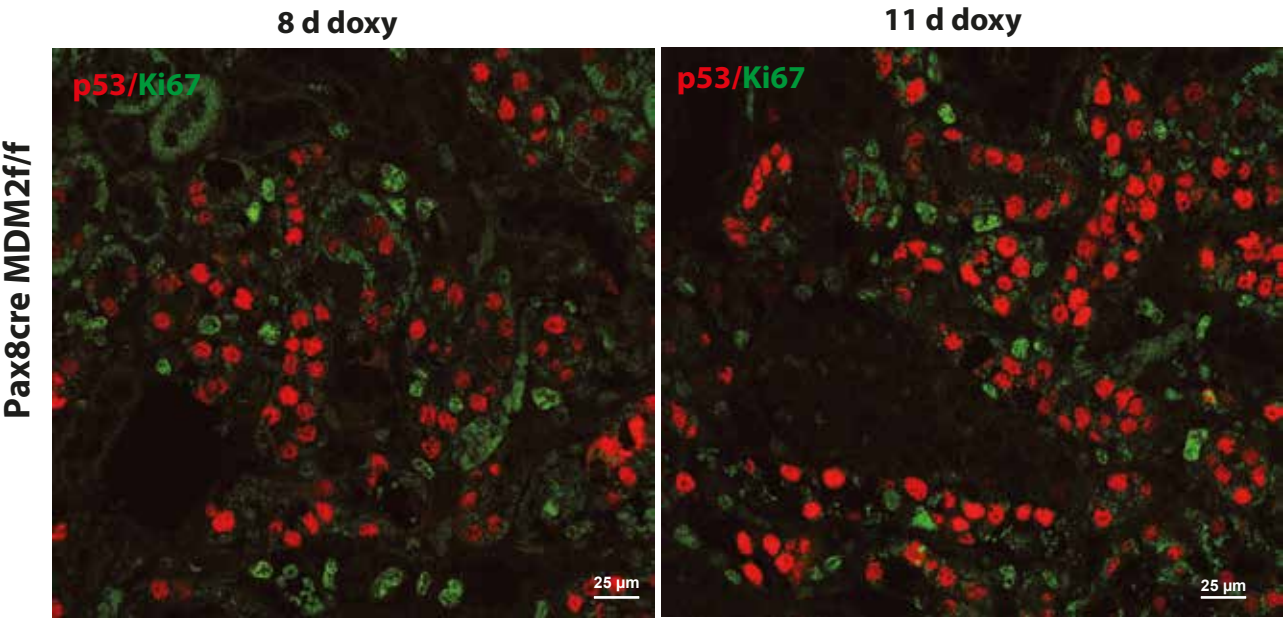

B

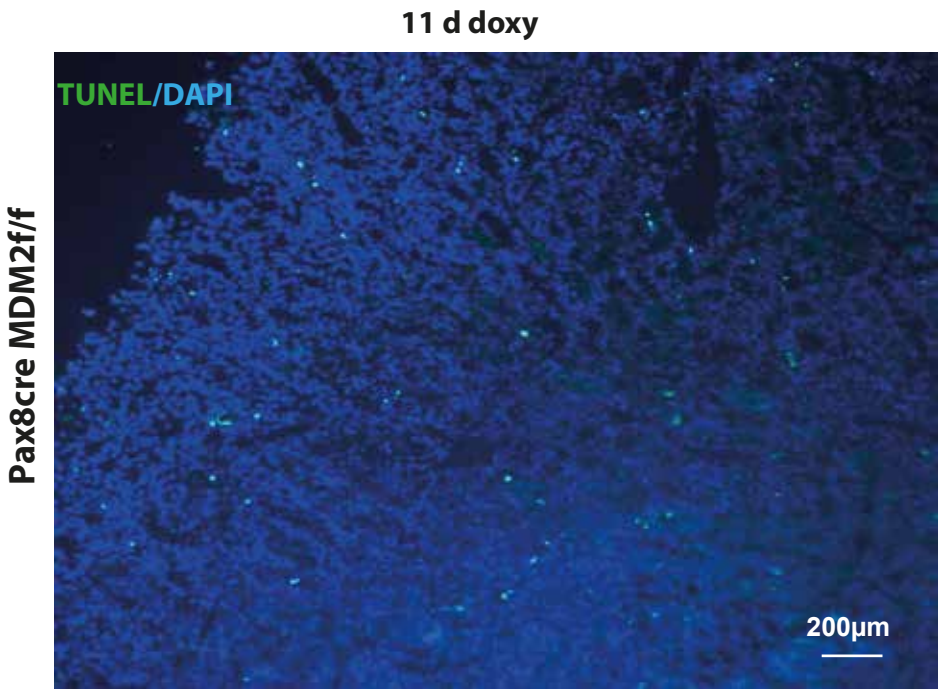

Supplement: Supplementary Figures [file cddis2016390x1.pdf]
