# Supplementary material for: MDM2 prevents spontaneous tubular epithelial cell death and acute kidney injury
Source: Cell Death Dis. 2016 Nov 24;7(11):e2482–. doi: 10.1038/cddis.2016.390 (PMC5260907; doi:10.1038/cddis.2016.390)
Supplement: Supplementary Information [file cddis2016390x5.doc]

**Supplementary figure 1.** A. Murine tubular epithelial cells were incubated for 24h with MDM2 siRNA or control scrambled siRNA and p21 and PUMA mRNA expression was measured by RT-PCR. B. Murine primary tubular cells isolated from *Pax8rtTA-cre;MDM2f/f* were treated with 1µg of doxycycline or medium for 24 hours, than we measured p21 and PUMA mRNA expression by RT-PCR. C. To proof the specificity of the MDM2 depletion in tubular epithelial cells, we isolated also glomerular cells from kidneys of *Pax8rtTA-cre; MDM2f/f*, treated them with doxycycline and analysed A. mRNA levels of MDM2 and B. viability of those cells. Unlike in primary tubular cells, the MDM2 expression and viability remained unchanged in the glomerular cells in comparison to the controls. Data are means ± SEM. ** p<0.01, ***p<0.005 .

**Supplementary figure 2.** A. MDM2 staining in healthy mouse kidney showing high expression of MDM2 in tubular cells and podocytes. Lectin tertragonolobus staining marks proximal tubules.

B. RT-PCR analysis confirmed highly elevated expression of p53 target gene p21 mRNA (60x elevated at day 8) and less increased (7x elevated at day 8) expression of PUMA mRNA in kidney lysates of *Pax8rtTA-cre;MDM2f/f* mice treated with doxycycline for 8 and 11 days. Data are means ± SEM. ***p<0.005 .

**Supplementary figure 3. A.** Representative images of kidney sections from *Pax8rtTA-cre;MDM2f/f* mice kidneys treated for 8 or 11 days with doxycycline showing co-staining for Ki-67 (green), marker of proliferation or hypertrophy and p53 (red), marker of the cell cycle arrest, senescence and cell death. We did not detect any co-locatization of Ki67 and p53 positive cells. The images are shown at a magnification of ×200. B. Representative image of TUNEL staining of kidney section from *Pax8rtTA-cre;MDM2f/f* mice kidneys treated for 11 days with doxycycline. The images are shown at a magnification of ×100.

**Supplementary Movies**

**Supplementary Movie 1** shows a 3D reconstruction from an *in vivo* 2-PM z-stack of a Tg(wt1b:eGFP) zebrafish larvae pronephros following the KD of mdm2 at 4 dpf. A significant dilatation of Bowman’s space can be seen as well as dilatation of the proximal tubule lumen.

**Supplementary Movie 2** shows a 3D reconstruction of the pronephros of a larva following the KD of mdm2/p53 with rescue of the phenotype seen in supplementary movie 1.

**Supplementary Movie 3** shows a 3D reconstruction of the glomerulus and proximal tubules of a Ctrl larva with normal pronephric morphology.**shhishihs**
